# Cascade training for scaling up care for perinatal depression in primary care in Nigeria

**DOI:** 10.1186/s13033-023-00607-5

**Published:** 2023-11-20

**Authors:** Bibilola D. Oladeji, Olatunde O. Ayinde, Toyin Bello, Lola Kola, Neda Faregh, Jibril Abdulmalik, Phyllis Zelkowitz, Soraya Seedat, Oye Gureje

**Affiliations:** 1 Department of Psychiatry of Psychiatry, College of Medicine, University of Ibadan, Ibadan, Nigeria; 2 WHO Collaborating Centre for Research and Training in Mental Health, Neurosciences and Drug and Alcohol Abuse, Department of Psychiatry, College of Medicine, University of Ibadan, Ibadan, Nigeria; 3 Department of Psychology, Carleton University, Ottawa, ON Canada; 4 McGill University, Montreal, Canada; 5 Department of Psychiatry, Faculty of Medicine and Health Sciences, Stellenbosch University, Cape Town, South Africa

**Keywords:** Cascade Training, Task-sharing, Primary healthcare, Low- and middle-income countries, Perinatal depression

## Abstract

**Background:**

Task-shared care is a demonstrated approach for integrating mental health into maternal and child healthcare (MCH) services. Training and continued support for frontline providers is key to the success of task sharing initiatives. In most settings this is provided by mental health specialists. However, in resource constrained settings where specialists are in short supply, there is a need to explore alternative models for providing training and supportive supervision to frontline maternal care providers. This paper reports on the impact of a cascade training (train-the-trainers) approach in improving the knowledge and attitudes of primary healthcare workers (PHCW) to perinatal depression.

**Methods:**

Senior primary health care providers selected from across participating local government areas were trained to provide training to other PHCWs. The training sessions facilitated by these trainers were observed and rated for fidelity by specialist trainers, while the trainees provided their impression of and satisfaction with the training sessions using predesigned assessment forms. Training outcomes assessed included knowledge of depression (using mhGAP training questions and knowledge of depression questionnaire) and attitude towards providing care for depression (revised depression attitude questionnaire (R-DAQ)) measured pre and post training as well as six months after training.

**Results:**

Trainees were 198 PHCWs (94.4% female), who routinely provide MCH services in 28 selected primary care clinics and had between 6- and 34-years’ experience. Training was provided by 11 trained trainers who were general physicians or senior nurses. Training sessions were rated high in fidelity and on training style. Sessions were rated excellent by 77.8% of the trainees with the trainers described as knowledgeable, effective and engaging. Knowledge of depression mean score improved from a pre-training level of 12.3 ± 3.5 to 15.4 ± 3.7, immediately post-training and 14.7 ± 3.2, six months post-training (both comparisons: p < 0.001). The proportion of PHCW workers endorsing statements indicative of positive attitudes on the professional confidence and the generalist perspective modules of the R-DAQ also increased with training.

**Conclusion:**

Our findings suggest that cascade training can be an effective model for rapidly providing training and upskilling frontline PHCWs to deliver care for women with perinatal depression in resource limited settings.

**Trial registration:**

This study was retrospectively registered 03 December 2019. 10.1186/ISRCTN 94,230,307.

## Introduction

Depression is the leading cause of disease burden in women of childbearing age globally [1]. The prevalence of perinatal depressive disorder (that is depression occurring during pregnancy and continuing or beginning in the first year postpartum) ranges from 10 to 15% in high income countries [2, 3]. The rates are higher in low and middle-income countries where the estimates are between 16 and 20% [4, 5]. Studies from Nigeria report that between 8% and 30% of perinatal women have depression [6, 7]. Perinatal depression has a negative impact on obstetric outcomes, infant development, and mother-child interactions. Contributing to the disease burden is the observation that perinatal women with depression are less likely to have their mental health needs met [8]. One major reason for this treatment gap is that most health providers who attend to women in the perinatal period are unable to identify the disorder. Estimates in some high-income countries suggest that less than 50% of cases of postnatal depression are detected by primary health care professionals in routine clinical practice [9]. In low- and middle-income countries where primary care is commonly provided by minimally trained primary health care workers rates are likely to be much lower. In an earlier report in Nigeria only 3 out of 218 (1.4%) pregnant women who screened positive for depression (had a score of 12 or more on the Edinburg Postnatal Depression Scale) were identified as having depression (that is, had a diagnosis of depression documented in their clinic records) at first contact with primary healthcare workers [10].

There is evidence from low- and middle-income countries (LMIC) that the negative consequences of perinatal depression can be reduced through mental health interventions delivered by supervised non-specialist healthcare providers. A systematic review and meta-analysis by Rahman et al. [11] reported that psychosocial interventions delivered by non-specialist health and community workers proved to be more beneficial than routine care at reducing the symptoms of depression, improving mother–infant interactions and infant outcomes, such as cognitive development and growth, reducing diarrhea episodes and increasing immunization rates. Studies are now needed to improve our understanding of how these interventions can be scaled up across settings in LMIC that may be highly diverse.

In recent years, task-sharing initiatives have been shown to be a viable option for integrating mental health care into primary maternal and child healthcare [11]. The success of task sharing initiatives hinges on the training and supervision of non-specialist providers who are at the forefront of delivering care. Studies have demonstrated that with training and ongoing supervision, non-physician primary health care providers can identify common perinatal mental disorders and deliver effective interventions [8, 12]. However, given that mental health specialists are few in most LMICs and are often overwhelmed by the demands of providing services, they may lack the additional time required to provide the needed training and supervision of frontline primary care workers to improve mental health service delivery. A cascade training program in which a small group of senior primary care providers are trained to become trainers who are then able to transfer relevant knowledge and skills to their peers may be a more feasible and sustainable alternative [13]. This model has the potential for rapidly upskilling the workforce.

In an earlier study, our team demonstrated the effectiveness of a cascade training format in which mental health specialists (designated as Master Trainers) trained non-specialist physicians and senior nurses (Trainers or Facilitators) to further train other frontline primary care providers to recognize and care for mental disorders using the WHO Mental Health Gap Action Programme -Intervention Guide (mhGAP-IG) [14]. Over the course of 12 months post-training, there was an average increase of 400% in the proportion of patients attending the clinics who received a mental, neurological or substance abuse (MNS) diagnosis [15]. Evidence for the utility of this approach for scaling up care for perinatal mental health problems in routine primary care especially in resource constrained settings is needed.

The mhGAP-IG is specifically designed to be used by non-specialists working in primary care to scale up mental health service in LMIC [16]. While the mhGAP provides evidence-based guidelines, implementation depends on country context as there are differences across countries in health care system structures, training and qualifications of frontline providers expected to implement the guidelines, as well as the availability of modalities of treatment for different conditions. The mhGAP has been contextualized for the Nigerian health care system [14] and was adopted by the highest decision-making body for health (National Council on Health) as a tool to scale up mental health care in Nigeria.

Bridging the gap between research evidence and practice requires the use of an effective implementation strategy or framework. The Replicating Effective Programs (REP) Framework was adopted for this current study as it provides a roadmap for implementing evidence-based interventions into community settings through a combination of intervention packaging, training, technical assistance, and other strategies to maximize the chances for sustainability [17]. The REP framework outlines strategies for maintaining treatment fidelity while identifying opportunities for tailoring the intervention to fit local needs (Kilbourne et al., 2007). The REP was initially developed by the US Centers for Disease Control and Prevention to package and disseminate HIV behavioral and treatment interventions for implementation in community-based service settings [18]. There are four phases to the REP framework: (1) Precondition- main goal of this phase is to identify the need, identify effective interventions, barriers and to draft an intervention package; (2) Pre-implementation- during this phase, with the help of a community working group, the package is further developed, the training and technical assistance needed is identified, pilot testing is conducted, and the final package is prepared; (3) Implementation phase involves the training of selected staff from the participating organizations, implementing the technical assistance components and evaluating the outcomes; (4) Maintenance and evolution phase involves organizational changes to sustain the intervention.

A major limitation of previous implementation studies using task sharing approach is the limited information available on training and its outcomes to show what works and what does not to assist in decisions about replicating a particular training approach [19, 20]. Here, we describe the implementation of a cascade training program for scaling up of care for perinatal depression in primary care in Nigeria using the Replicating Effective Program (REP) framework. We also report on changes in knowledge and attitudes of frontline primary healthcare workers before and after training.

## Methods

The study - Scaling up care for perinatal depression for improved maternal and childcare (SPECTRA) is a type-II hybrid implementation study. SPECTRA was conducted in primary healthcare centres selected from across 11 local government areas located within and around the city of Ibadan, the capital of Oyo State, Nigeria between 12 August 2015 to 11 February 2020. The study adopted a before and after design to explore both implementation and effectiveness outcomes. The primary implementation outcome was defined as change in the rate of identification of perinatal depression among the providers and the effectiveness outcome was remission rates of depression at 6 months postpartum between the two patient cohorts; the first cohort was recruited before training PHCW and the second cohort after the training [21].

### Study setting and selection of PHCCs

**Fig. 1:**
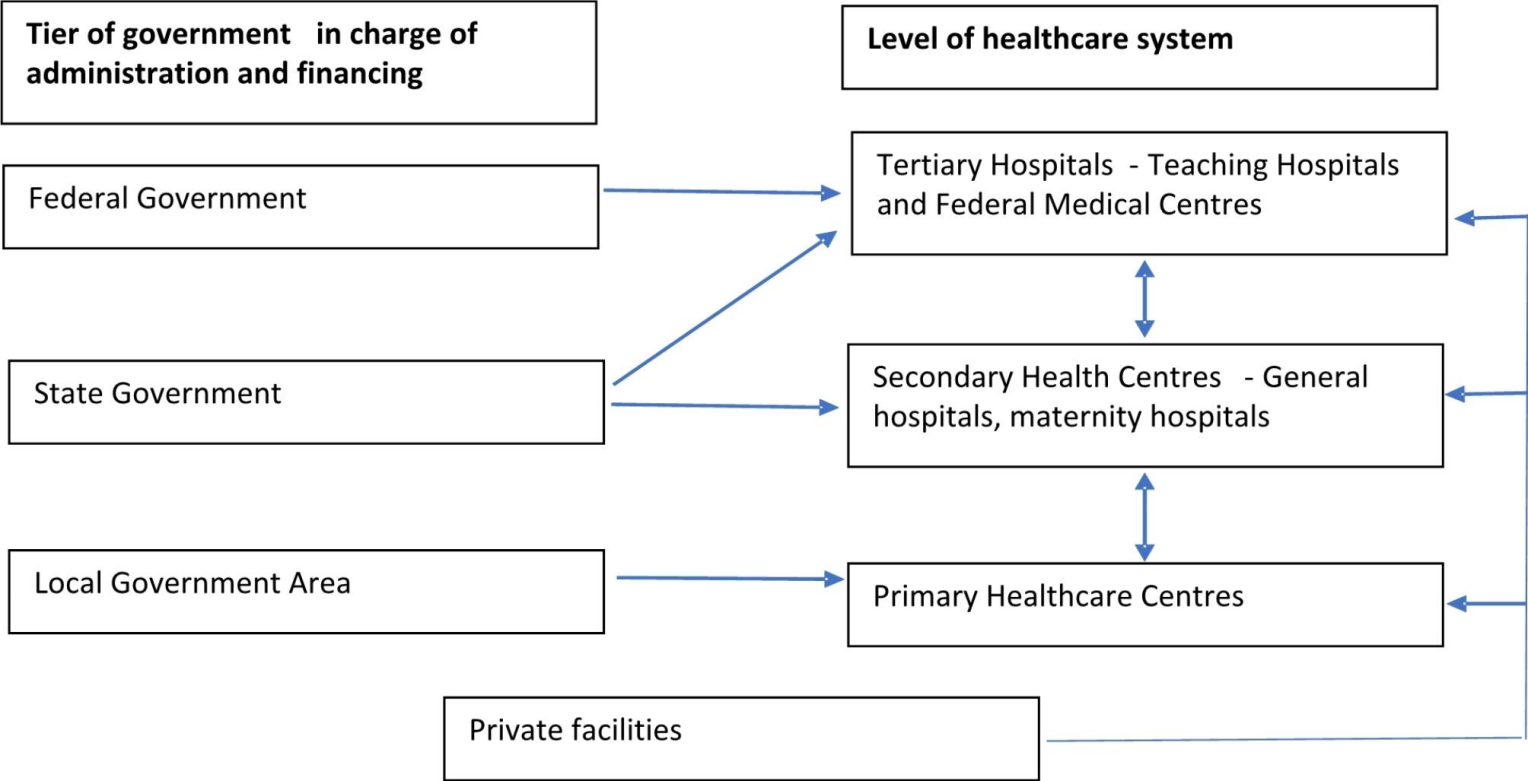
Healthcare System in Nigeria

The health system in Nigeria is 3-tiered - primary, secondary and tertiary (Fig. 1). The primary care level is the entry point into the health system and comprises government run primary health care centers, private clinics as well as general clinics of general and teaching hospitals. The government run primary healthcare system has 3 different types of facilities (the primary healthcare center, the primary health clinics and health posts) distinguished by their structure, staffing, equipment and available services. The setting for this study is the primary healthcare center (PHCC). The PHCC has a full complement of staff (nurse/midwives, community health officers (CHO) and community health extension workers (CHEW)) and deliver the full range of services expected at the primary care level. There are about 10–14 PHCCs in each local government with each headed by the most senior provider in the facility (nurse/midwife, CHO or CHEW). In Oyo State where this study was carried out, the group of primary health care facilities in each local government is supervised by a primary healthcare coordinator who is usually a physician, who oversees the services provided across all the facilities in the local government.

A mapping exercise was carried out to assess the profile of the clinics in terms of their patient flow, complement of primary care workers available and the services rendered. A total of 132 clinics are located within the study area, 76 clinics were initially excluded (25 were actively engaged in other ongoing trials and were unavailable; 29 did not have an adequate complement of staff; 12 did not provide a full range of perinatal service; 10 did not provide consent). An additional 18 clinics were excluded because these clinics were part of the high intensity treatment arm of our earlier randomized controlled trial of depression in primary care [7] and their staff had therefore received prior training on mental health and delivery of more structured psychosocial interventions. Twenty-eight of the remaining 38 available clinics were included in this study.

**Fig. 2:**
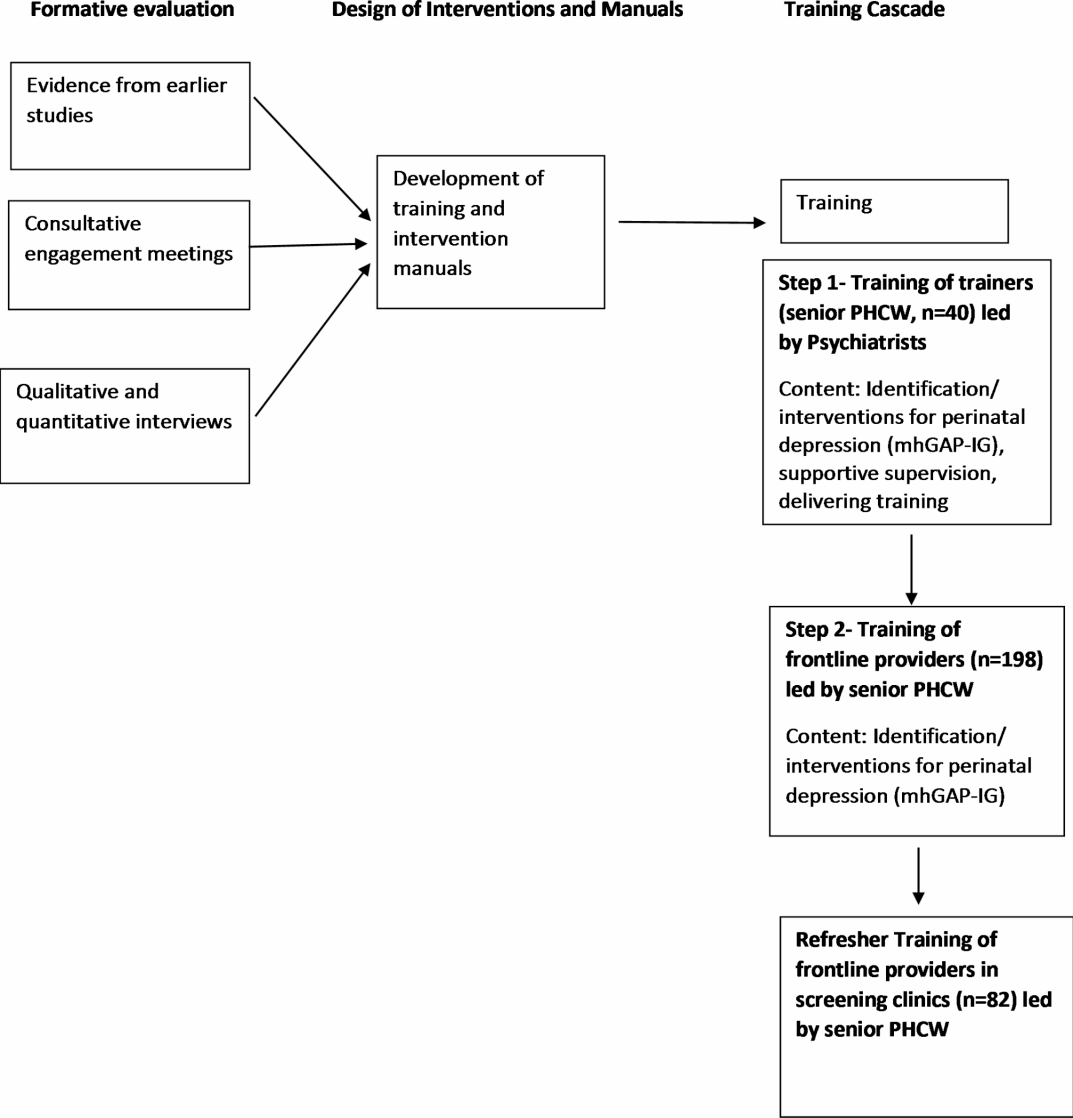
Design of the cascade training

In line with the REP framework, the study was conducted in four phases with relevant activities planned for each phase (see Table 1). Only the procedures and activities relevant to the cascade training are reported here. Table 1 provides an overview of the components of the REP as applied in this study and Fig. 2 shows the design of the training.

**Table 1:** Replicating Effective framework for the implementation of cascade training for perinatal depression in primary healthcare in Nigeria

REP Component	REP Elements	Program Activities	Decision/ outcome
Pre-conditions	Identify effective intervention Identify barriers to implementation of training	-Review of findings from earlier trials -Stakeholder meetings -Qualitative interviews with clinic heads- assessment of the organization structure of the clinics, training needs -Planning workshop	-More feasible to use the mhGAP-IG interventions used in the low-intensity-intervention of earlier trial -Stagger training for PHCW in same clinic so as not disrupt service -Training venue outside of clinics to reduce distractions
Pre-implementation	Draft intervention package	Development of training manuals Development of intervention manuals Development of support tools	-Training manual to include all components of the intervention manual, the powerpoint slides and training module. -Manual needs to be brief and in simple language -Desktop chart to guide assessment -Make available abridged version of the mhGAP-IG containing relevant modules to PHCW - Supportive supervision checklist
Implementation	Training of trainers Training of frontline providers Evaluation Technical assistance	Interactive training workshops facilitated by master trainers (psychiatrists) Interactive workshops- didactic teachings, group work and role plays -Pre and post test questions at each workshop -Satisfaction with training -Fidelity to the delivery of the training sessions -Training on the use of PHQ-2 Supportive supervision	Selection of trainers to facilitate the training workshops from among the trained trainers All frontline PHCWs in all selected clinics trained in batches Half of the clinics randomized to use the PHQ-2 for screening Trainers visited clinics monthly to provide support and reported to the research team
Maintenance and Evolution	Feedback and refinement	-Retention of knowledge evaluated 6 months after training -Debriefing -Refresher trainings	Same questionnaire used for the pretest administered -Group discussions held before each refresher training workshop -Training with emphasis on areas of difficulty

### Phase 1 pre-condition

In an earlier trial in primary healthcare clinics in Nigeria, we demonstrated that a low intensity intervention based on the mhGAP-IG recommendations was effective for managing perinatal depression [7]. This intervention was therefore adopted for scale-up in the SPECTRA study. The precondition phase was focused on identifying the potential barriers and facilitators for scaling up this evidence-based intervention for perinatal depression. Consultative engagement and planning meetings were held with key stakeholders to secure their cooperation, explore their understanding of perinatal depression and how it is managed, in addition to obtaining their views on what is required for successful implementation of the training and intervention package. The stakeholders included: community leaders, policy makers, primary care providers and users of perinatal mental health services.

### Phase 2 pre-implementation

We next assessed the organizational context of the clinics: mapping the staffing structure, the capacity to implement a chronic care model, availability of in-service training and the provision of ongoing supervision. This information was obtained in qualitative interviews with the head of the PHC facilities and supplemented by quantitative interviews using both the patient and provider versions of the Assessment of Chronic Illness Care (ACIC) tool [22]. To provide baseline information about the rate of detection of perinatal depression and document the treatment that was currently provided for the condition, we recruited and followed up a cohort of women with perinatal depression from the selected PHCC. Further details of the methods as well as the results of this phase are provided in earlier publications [10, 21].

#### Development of training manuals and other training materials

Using information obtained from the earlier phase of the study, the mhGAP recommended interventions for perinatal depression were next adapted for delivery in primary healthcare. Key considerations were the understaffing of the PHCCs and the high patient load in the clinics necessitating that screening measures and interventions chosen had to be brief. This informed the choice of the 2-item patient health questionnaire (PHQ-2) for routine screening for perinatal depression as well as the use of the simple psychosocial interventions included in the mhGAP-IG (psychoeducation, encouraging activity, social network reactivation and support to resolve psychosocial stressors).

Two sets of manuals were developed- one for the trainers (training manual) and the other for the frontline providers (intervention manual). The intervention manual was brief outlining key points needed for the assessment of perinatal depression and basic psychosocial intervention skills. The training manual contained all the contents of the intervention manual, along with a module on how to provide training. It also included the power point slides that we earlier developed to facilitate training. All the PHCW were given copies of the mhGAP-IG depression module to facilitate history taking and to provide information about treatment guidelines. The intervention manual was designed to be used along with the mhGAP-IG depression module.

### Phase 3 implementation

#### Training of trainers

The primary health care coordinators along with three of the most senior primary healthcare providers (identified by the PHC coordinator) from each of the local government areas were selected to be trained as trainers. Invitation letters were sent out to the selected trainers across the eleven local government areas. Two training workshops were conducted for the trainers facilitated by two psychiatrists (Master Trainers). The format for these workshops was specifically designed to mirror the training for the frontline health workers that the trained facilitators were expected to conduct.

#### Training of frontline providers

All the frontline providers at all the selected clinics were invited for training. The training workshops were held in batches of 20–25 participants. To ensure that clinical services were not disrupted, not more than half of the PHCW in a clinic was invited at any given time for a workshop, leaving the other half to provide services.

#### Training workshops

Training workshops were organized outside of the primary care clinics in a conducive conference room with each workshop lasting 3 full days. The workshop adopted a blended learning approach using a combination of didactic interactive teaching sessions aided with power point slides/ flip charts, discussion sessions and role plays. The content of the teaching sessions was divided into 9 modules- overview of the mhGAP-IG, depression and how to make a diagnosis, perinatal depression, depression and suicide, basic counseling skills, psychosocial interventions for depression (psychoeducation, how to support a patient to address current psychosocial stressors, reactivating social network). There were practical discussion sessions focused on each lecture topic. There were 3 role plays– the first role play focused on the use the mhGAP-IG to guide the assessment of signs and symptoms of depression and making a clinical diagnosis of depression. The second role play was to practice psychoeducation, supporting patients to identify and address current psychosocial stressors and reactivation of social network while the third role play was on decision-making for referral to a physician. PHCW attendees at the workshop were divided into groups of 3–4 for the role plays; and they took turns to play the role of a health worker, patient or patient relative and observer (to provide feedback to the health worker and discuss ways to improve on the skills). A debriefing session followed each role play to discuss the observations, and areas of difficulties.

#### Training on the use of the 2-item patient health questionnaire (PHQ-2)

The selected clinics for the study were then stratified based on patient load and randomly assigned to arms: screening and non-screening . The clinics assigned to the screening arm were visited by one of the psychiatrists (master trainers) who delivered training on how to administer the PHQ-2 onsite. Copies of the PHQ-2 were attached to the clinic paper records of all antenatal women registering in these clinics for the use of the PHCW during triage. In addition, the screening clinics were scheduled for booster training to enable an exploration of the effect of these interventions on identification and care provided for perinatal depression.

### Outcome assessments

The implementation outcomes collected for the training included fidelity of the trainers to delivering the training sessions during the stepdown training, their training style and the satisfaction of the trainees with the training (acceptability). The fidelity to training and training styles were rated by specialist trainers sitting in during the training sessions while satisfaction was rated by the trainees. The intervention outcomes included change in the frontline providers (trainees) knowledge of perinatal depression and their attitude towards providing care for depression as well as their overall impression of the training they received.

*Fidelity*: The fidelity to training specifications was scored using a form specifically designed for this purpose. Each of the 3-day training workshops were facilitated by 2 of the trained trainers who allocated the modules amongst themselves. Master Trainers (psychiatrists) sat in on training sessions and rated the trainers on content fidelity and training style as well as document other observations. Each topic under the 9 modules expected to be covered by the trainers during the training sessions were rated on a three-point scale: (1) Satisfactory coverage, (2) Partial Coverage, (3) Poor coverage. Training style was rated on 3 relevant areas i) Body language (eye contact, vocal projection, inflection and clarity, use of purposeful gestures); II) Use of slides (clear transition from topic to topic, organization, appropriate vocabulary and explanations); and iii) Time management (punctuality, keeping to the time allotted for each session).

*Satisfaction with training* was reported using a series of open-ended questions administered to the PHCWs after the last session of the training. It explored their overall satisfaction with the training and the organization of the workshops, what they liked most, what they did not like about the training and their suggestions for improvement. Overall satisfaction with the training programme was rated on a 5-point scale- poor, fair, satisfactory, very good and excellent.

#### Knowledge of Depression

This was assessed using a combination of mhGAP training questions and an adapted version of the knowledge of depression questionnaire [23]. The knowledge of depression questionnaire consists of 27 multiple choice questions. For our study we omitted questions related to medications and electroconvulsive treatment and modified some of the questions to focus on perinatal depression. In all we used 7 items from the mhGAP training pre-test questions, 16-items based on the knowledge of depression questionnaire, and we included four additional questions to assess other areas of the training (such as psychosocial interventions) not covered by the selected instruments. The questions were in the multiple choice single answer format, with the total scores ranging from a minimum of 0 to a maximum of 27.

#### Attitude to depression

This was assessed using the revised depression attitude questionnaire (RDAQ) (Haddad et al., 2015). The RDAQ is a 22-item questionnaire scored on a 5-point likert scale indicating level of agreement to statements assessing clinicians’ attitudes to depression. The R-DAQ was developed in the UK to assess the attitudes of general practitioners (GPs) towards depression and taking care of patients with depression (Haddad et al., 2015). It has been used in other settings among other categories of clinicians (Haddad et al., 2016). Three subscales of the R-DAQ were identified in earlier use categorizing clinicians’ attitudes about (1) professional confidence in depression care, (2) therapeutic optimism/pessimism in depression care, and (3) general perspectives about depression occurrence, recognition and management. Here we report the proportion of providers endorsing each item (agree/ strongly agree).

To provide an assessment of the effect of the training on the knowledge of depression and attitudes towards providing services for perinatal depression, the knowledge of depression questions and the RDAQ were administered before and after the training sessions to all participants.

### Phase 4: maintenance and evolution

Refresher Trainings: Refresher training for all the providers at the screening clinics were conducted by the trained trainers about 7 months after the initial training. The refresher trainings started with debriefing sessions to explore the experiences of the providers with delivering care and to identify the areas of deficiencies that needed more emphasis during the sessions.

Retention in knowledge and attitudes: The knowledge of the primary care providers working at the screening clinics about depression as well as their attitudes were reassessed about 6 months into the intervention program.

### Data analysis

Descriptive demographic data and response to the RDAQ are presented as numbers, means and percentages as appropriate. Repeated-measures ANOVA was used to explore the variation in the mean knowledge of depression scores across the three time points (pre-test, post-test and six months after training) on the subset of providers in the screening clinics who were assessed at all the time points. A post hoc pairwise comparison using the Bonferroni correction was used to assess the difference between the pre-test vs. posttest and the pretest vs. six-months post training mean knowledge of depression scores. The assumption of sphericity was also carried out by running the Mauchly’s test of sphericity and this was satisfied by our data. Factors associated with healthcare workers’ knowledge in the pre-test were explored by a regression analysis. The level of significance was set at p < 0.05. The responses of the providers to the open-ended questions were explored using thematic analysis. A mix of inductive (themes derived from the data) and theoretical (themes influenced by the literature) approaches were used.

## Results

### Training of trainers

Invitations were sent out to the primary health care coordinators and three of the most senior primary health care workers from each of the 11 local government areas in which the study was being implemented for training to form a pool of potential trainers (N = 44). Forty of these were available for the training of trainers’ workshops. Two workshops with 20 participants each were conducted. Training was provided by two psychiatrists. Senior level PHCWs trained included: 5 Doctors, 22 Nurse/midwives, 7 Community Health Officers, 6 Senior Community Health Extension Workers. Out of these, 12 that we considered to be the most competent (good communication skills, enthusiastic about mental health and had been part of earlier intervention studies), and with more years of experience working in primary care, were invited to facilitate the workshops for the frontline providers. However, one was not available at the time of the training workshops, hence 11 trainers facilitated the workshops for the other PHCWs.

#### Training of frontline providers

Each training workshop was facilitated by two of the trainers with at least one of the psychiatrists sitting in to observe the training and provide support. Eight training workshops with 20–24 participants in each were conducted. Trainees were all the primary healthcare workers in the selected clinics who routinely provided maternal and child healthcare services (n = 198). The ages of the providers ranged between 30 and 56 years, 187 (94.4%) were female and the number of years of experience in delivering care ranged from 6 to 34 years with a mean (sd) of 20.1 (6.9) years. About a third (37.9%) reported previous training in mental health care. (Table 2)

**Table 2:** Demographic characteristics of the frontline primary healthcare workers

Demographic characteristics (N = 198)	n (%)
**Gender**	
Male	8 (4.1)
female	190 (95.9)
**Cadre**	
Community Health Extension Worker (CHEW)	104 (52.5)
Community Health Officers (CHO)	55 (27.8)
Nurse/ midwives	28 (14.1)
Others	11 (5.5)
**Previous in-service mental health training**	
Yes	75 (37.9)
No	123 (62.1)
**Age**	
Mean (SD)	44.7 (6.5)
Min, Max	30, 56
**No of years in service**	
Mean (SD)	20.1 (6.9)
Min, Max	6, 34

### Fidelity of trainers to the training

Six out of the 8 training sessions (one session conducted by each of the 6 pairs of trainers) were rated for fidelity). Overall, 81.9% of the items on the 9 modules taught by the trainers were rated as being satisfactorily covered, 17.6% rated as being partially covered and 0.5% rated as poorly covered. The module- ‘what is depression/how to diagnose depression’ had the lowest proportion of its items (57.1%) rated as being satisfactorily covered while the module on ‘psychosocial treatment of depression’ had all items covered satisfactorily. A key observation by the master trainers was that the non-physician trainers had more difficulty with facilitating the module on depression and most of the partial or poor coverage ratings were for that module. All the facilitators met or exceeded expectations on the rating of all the different sections of the form used to assess training style.

### Satisfaction with training

Out of the 176 participants that rated their overall satisfaction with training, 137 (77.8%) rated the training as excellent, 28 (15.9%) rated as very good and 11 (6.3%) rated the training as satisfactory. No participant rated the training as fair or poor. The key themes that emerged from their responses to the open-ended questions exploring their views about the training (the mode of training, the facilitation of the training) and their likes and dislikes, and their suggestions for improving the training (selected quotes on each of the themes are provided in Table 3): (1) The modality for the training- the PHCW expressed satisfaction with the method of teaching, and the training materials used. (2) They also thought their trainers were knowledgeable and engaging. (3) They were delighted at the facilitators’ ability to communicate in the language that the providers could easily understand. (4) They expressed their satisfaction with the new skills and knowledge acquired. (5) The key theme that emerged on what they did not like about the training centered on the short duration (3 days), making the course quite intensive. (6) Their suggestions for improving the training revolved around the (i) need for refresher trainings with some PHCWs suggesting annual training; (ii) need for supervision and monitoring; and (iii) need to involve other sectors and get government to collaborate on efforts to scale up care for perinatal depression.

**Table 3:** Selected quotes from open-ended responses of the primary care workers on the training

Theme	Quotes
What they liked about the training	· Everything - Especially the roleplay, Kudos to the Organisers · Method of teaching, materials given, resource personnel · The way training was presented, will enable me to deal with depressed patient
Perception of the PHCW of trainers	· Loved their teaching, created rapport with us, gave answer on every question asked and (I) gained more knowledge on depression · Interesting, illustrative, and educating, increased my skill and knowledge · Facilitators did well, took time to teach us, I now know better on depression
Language used for training	· The training is very interesting, easy to understand · Clear communication between trainers and trainee, visual communication also, able to interact in our own language · Communicated in the language I understand, the facilitators are fantastic, good for providers · All our lecturers used our languages to teach us (Yoruba) and also maintain good interaction with us
Skills acquired during the training	· Gained knowledge on signs and symptoms of depression · PHC workers can now assess, decide and manage perinatal depression cases · The training is so interesting, my knowledge about depression and perinatal depression increased · Health worker can now treat depression by psychosocial intervention · I will be a good caregiver in assessing, diagnosis and management of any client with perinatal depression in my place of work
Dislikes about the training	· Course stressful · Duration is too short · Days of training too short
Suggestions for improving the training and moving forward	· Organise more trainings every 6 month or a year for everybody again · Programmes should not stop, it should be a continuous exercise · This training should cut across all health sectors and there should be monitoring from the trainers · Persistent monitoring of the health personnel to encourage them to impact the knowledge acquired by them to stepdown the training to the health workers at work · They should come for supervision all time and retrain us · I suggest that the training should be continuous so as to help all the health workers at Primary Health care level to improve in attending to people with mental disorders · Training should be extended to all health care providers in Oyo State PHC with state govt collaboration · Early intervention, more training for people on how to move closer with people, Government should please employ personnel, timely attention should be focused

### Knowledge of depression

The mean pre- and post-test scores of the primary healthcare workers who were assessed at all three time points (n = 80) on the knowledge of depression question was 12.3 (SD 3.5) and 15.4 (SD 3.7), respectively. Six months after the initial training, the mean score was 14.7 (SD 3.2). The mean knowledge of depression scores differed significantly across the three time points (F(2,162) = 33.336, p < 0.001). Compared to the pre-test mean, the means at post-test and six-months post-training were significantly higher (p < 0.001). While there was a slight drop between post-test and six-months post-training mean knowledge scores, this did not reach statistical significance (15.4 vs. 14.7, p = 0.313).

The factors that were associated with healthcare workers knowledge in the pre-test were exposure to earlier trainings on mental health and number of years in service (providers with shorter years in service scored higher on the knowledge test).

### Attitudes to depression

The proportion of PHCWs endorsing statements indicating positive attitudes on the professional confidence in depression care subscale of the R-DAQ increased with training (Table 4). The proportions of PHCWs endorsing positive attitudes increased further at six months post-training on items such as “my profession is well placed to assist patients with depression”, “I feel confident in assessing depression in patients” and “I feel comfortable in dealing with depressed patients’ needs”. There was a similar observation in the subscale assessing their perspectives about the occurrence, recognition and treatment of depression and some items on the therapeutic optimism/pessimism about depression. For example, the proportion of providers endorsing statements about lack of response to treatment (“depression reflects a response which is not amenable to change” and “once a person has made up their minds about taking their own life no one can stop them”) decreased following training and further reduced at six-months post-training. The proportion of providers with negative views about the cause of depression was generally low in this sample of providers. However, while the proportion endorsing these views reduced immediately following the training, by the six months follow up assessment the proportion had increased (Details in Table 4).

**Table 4:** Attitudes towards depression

Depression attitudes R-DAQ items	Pre-test (N-198)	Post-test (N-198)	6-months post-training (N-80)
*Professional confidence in depression care*	n (%)	N (%)	N (%)
It is rewarding to spend time looking after depressed patients	138 (81.7)	163 (83.6)	65 (83.3)
I am more comfortable working with physical illness than with mental illness like depression	63 (36.0)	40 (20.6)	21 (26.6)
My profession is well placed to assist patients with depression	158 (88.3)	171 (87.7)	79 (97.5)
I feel confident in assessing depression in patients	139 (78.1)	168 (86.6)	80 (100.0)
I feel comfortable in dealing with depressed patients’ needs	144 (78.3)	174 (88.8)	77 (96.3)
My profession is well trained to assist patients with depression	148 (84.6)	175 (88.4)	77 (95.1)
I feel confident in assessing suicide risk in patients presenting with depression	109 (64.9)	166 (86.5)	68 (85.0)
***Generalist perspective about depression occurrence, recognition, and management***			
Anyone can suffer from depression	144 (85.2)	173 (88.7)	76 (93.8)
All health professionals should have skills in recognising and managing depression	157 (89.2)	167 (86.1)	77 (97.5)
Recognising and managing depression is often an important part of managing other health problems	144 (84.7)	174 (88.8)	77 (96.3)
People with depression have care needs similar to other medical conditions like diabetes, COPD or arthritis	112 (65.9)	151 (78.2)	66 (83.5)
Depression is a disease like any other (e.g. asthma, diabetes)	64 (35.8)	133 (68.6)	46 (57.5)
**Therapeutic Optimism**			
One of the main causes of depression is a lack of self-discipline and will-power	45 (25.6)	31 (15.8)	22 (27.5)
Becoming depressed is a way that people with poor stamina deal with life difficulties	66 (38.8)	61(31.9)	32(40.5)
Becoming depressed is a natural part of being old	49 (27.4)	52 (26.9)	24 (32.0)
Once a person has made up their mind about taking their own life no one can stop them	57 (33.1)	42 (21.6)	15 (19.0)
Depression treatments medicalise unhappiness	49 (27.8)	54 (27.7)	30 (39.0)
Becoming depressed is a natural part of adolescence	46 (27.2)	46 (23.8)	18 (22.8)
Depression reflects a response which is not amenable to change	56 (33.5)	41 (21.9)	13 (16.9)
There is little to be offered to depressed patients who do not respond to initial treatments	60 (36.4)	52 (26.8)	23 (28.8)
Psychological therapy tends to be unsuccessful with people who are depressed	52 (29.1)	43 (22.5)	19 (24.7)

## Discussion

Building the capacity of frontline providers through training, re-training, and supportive supervision is the bedrock to successful implementation of task-shared intervention programs. This report provides an overview of the implementation of a cascade training program for perinatal depression in a low-resource setting. The results from our study suggest that with support, trained senior non-specialist healthcare workers can effectively provide training to other frontline primary healthcare workers for perinatal depression with good fidelity. The training provided by the trained trainers resulted in significant improvement in the frontline workers’ knowledge of perinatal depression and psychosocial interventions, as well as improvements in their attitude and confidence towards rendering care for perinatal depression. The improvement in knowledge was noted immediately following the training and the slight drift in knowledge at the six-months post-training assessment was statistically non-significant, while the significant improvement over the baseline score was maintained.

Cascade training is an efficient and effective approach to address the shortage of trained healthcare workers in LMICs [13, 24]. This is particularly relevant for mental healthcare in the context of most LMICs where there is a pronounced shortage of human resources for mental healthcare. Nigeria like many other LMICs has very few psychiatrists available to the population. The WHO mental health atlas reports a median rate of psychiatrists to 100,000 of the population as 0.1 for low-income countries and 0.4 for lower middle-income countries compared to 8.6 in high income countries. The migration of skilled human resources including psychiatrists from LMICs to HICs contributes significantly to this scarcity of specialists in LMICs [25]. Across most LMICs, specialists to conduct direct training for frontline health workers are in short supply, so a cascade training approach could be a sustainable alternative in the interim.

Adopting a cascade training approach where the few available specialists train and support senior experienced physician and non-physician primary care providers to train other frontline providers may not just be feasible but likely to be the most viable, effective, and sustainable option for incorporating mental health services in primary care in low-resource settings. Our findings support the feasibility and effectiveness of cascade training for rapidly up-skilling frontline non-physician primary care providers to deliver care for perinatal depression. In this study, the effectiveness of this approach is supported by the improvements in the knowledge of depression of the PHCWs following training. The knowledge acquired by the PHCWs following training was sustained over time, albeit with a slight drift at the six months post training assessment. Similarly, the attitudes of the primary care workers towards treating depression improved immediately following the training with further improvement in some items during the repeat assessment at 6 months post training. The sustained improvement on items measuring professional confidence in depression care is a likely indicator that the providers put the skills acquired during the training to practice and became more adept at depression care. Prior to training, providers with shorter years in service had significantly higher scores on the knowledge test. While we did not explore the reasons for this, it might be a reflection that the more recently trained providers had increased exposure to mental health training.

This modality of training was acceptable to the PHCWs and they rated the training they received from the facilitators as very good or excellent. They felt the facilitators took their time to explain, communicated clearly and in a language that they could easily understand and were able to adequately address the questions asked. This advantage of cascade training has been reported in earlier studies; local trainers selected from the target population, are more familiar with contextual issues that may allow them to tailor the training to the needs of the trainees [26]. A notable observation was that in the fidelity ratings, the module on depression and how to diagnose depression had a higher proportion of items rated as poor or partial coverage especially with non-physician trainers during the didactic sessions. This suggests that in future trainings, pairing a physician with a non-physician trainer may be ideal to handle potentially difficult concepts in some modules.

Cascade training runs the risk of dilution and misinterpretation of the key message compared to direct training by specialist. However, studies suggest that this is addressed by reducing the number of training levels, using a blended learning approach (combining different techniques such as interactive, multifaceted methods and accompanying learning materials) and including monitoring and supervision during the training cycle [27, 28]. All these were taken into consideration in this study. The train-the-trainer workshops were organized to mirror the step-down trainings, the training combined different teaching techniques and the Master Trainers were present in the training workshop to support the trainers. The main drawback of multicomponent packages of training is the associated time, cost and intensity. This was evident in our study where the short duration of the training and intensity of the workshops emerged as the key themes in response to open-ended questions asking the PHCWs about what they disliked in the training sessions. However, considering the busy schedule of the providers and the need to maintain services during the training, increasing the number of days for the training may not always be feasible.

The importance of booster trainings and supportive supervision to the adoption and sustainability of task shared interventions in primary health care is well known. We observed that the knowledge of the primary care providers at the 6 months post-training re-assessment had started to decline. This suggests this might be a good time for booster training. This recommendation was reiterated by the PHCWs in response to open ended questions asking for their suggestions on how to improve the training.

While this study has a number of strengths including predetermined structured assessments of training outcomes and the introduction of measures to reduce the risk of dilution of the step-down trainings, the limitations are worth mentioning. The impact of the training was assessed immediately following training and at 6 months after. Some authors have argued that evaluation of the maintenance of training gains and treatment sustainability should be conducted 2 years following training. Also, while we demonstrated a change in knowledge and its sustainment at 6 months post training, it is unclear whether this translated into the use of the skill set in daily clinical practice and/or whether it resulted in improved patient outcomes. These are areas in need of exploration in future studies. Notwithstanding the success of this cascade training approach in building the capacity of primary care providers for maternal mental health, there is a need for LMICs to make mental health a priority through adequate financing as well as improving the availability of human resources for mental health. This require LMICs to invest in training and strategies for retaining mental health specialists (this could include innovative financial incentive packages, career building opportunities and improved working conditions) to support task sharing initiatives and improve access to evidence-based mental healthcare [29].

## Conclusion

The findings from this study suggest that adopting a cascade training model can be a viable option for providing training to large numbers of frontline primary healthcare providers in low resource settings where specialists are few. It would however be important to ensure that measures to overcome the limitations associated with cascade training are implemented alongside the training.

## Data Availability

The datasets generated and/or analyzed during the current study are not publicly available yet but are available from the most senior author on reasonable request.
